# Advances in the molecular characterization of multiple myeloma and mechanism of therapeutic resistance

**DOI:** 10.3389/fonc.2022.1020011

**Published:** 2022-10-27

**Authors:** Mateo Mejia Saldarriaga, Walaa Darwiche, David Jayabalan, Jorge Monge, Cara Rosenbaum, Roger N. Pearse, Ruben Niesvizky, Mark Bustoros

**Affiliations:** Division of Hematology and Medical Oncology, Department of Medicine, Weill Cornell Medicine, New York, NY, United States

**Keywords:** myeloma and other plasma cell dyscrasias, resistance, molecular subclassification, smoldering myeloma (SMM), MGUS, risk stratification

## Abstract

Recent insight in the genomic landscape of newly diagnosed multiple myeloma (NDMM) and its precursor conditions, monoclonal gammopathy of uncertain significance (MGUS), and smoldering myeloma have allowed the identification of patients with precursor conditions with a high risk of progression. These cases with “progressor” MGUS/SMM have a higher average mutation burden, have higher rates of mutations in specific genes such as MAPK, DNA repair, *MYC*, *DIS3*, and are enriched for specific mutational signatures when compared to non-progressors and are comparable to those found in NDMM. The highly preserved clonal heterogeneity seen upon progression of SMM, combined with the importance of these early variables, suggests that the identification of progressors based on these findings could complement and enhance the currently available clinical models based on tumor burden. Mechanisms leading to relapse/refractory multiple myeloma (RRMM) are of clinical interest given worse overall survival in this population. An Increased mutational burden is seen in patients with RRMM when compared to NDMM, however, there is evidence of branching evolution with many of these mutations being present at the subclonal level. Likewise, alterations in proteins associated with proteosome inhibitor and immunomodulatory drugs activity could partially explain clinical resistance to these agents. Evidence of chromosomal events leading to copy number changes is seen, with the presence of *TP53* deletion, mutation, or a combination of both being present in many cases. Additional chromosomal events such as 1q gain and amplification may also interact and lead to resistance.

## Introduction

Multiple Myeloma (MM) represents 1.8% of all new cancers in the United States (US) and is the second most common hematologic malignancy in the US with roughly 34,000 new cases/year (1), with a higher incidence and earlier age of diagnosis in African American (2 fold increase), while Asian population have a lower incidence (2–4). Advances in understanding of MM biology have led to therapeutic improvements over the last two decades, with over 10 new molecules targeting novel pathways and mechanism of action, and with a significant improvement in overall survival and quality of life since the introduction of these agents into clinical practice (5, 6).

MM is preceded by monoclonal gammopathy of undetermined significance (MGUS) and smoldering myeloma (SMM), collectively known as precursor conditions. More recently, it became clear MGUS/SMM precede the appearance of MM by several years (7, 8), representing a precursor state mirroring other hematological malignancies such as monoclonal B cell lymphocytosis and clonal hematopoiesis of indeterminate potential. Similarly, to MM, MGUS incidence is higher and at an earlier age in African American when compared to white population, suggesting the increase rate of MM seen in this population is the product of increased incidence of MGUS (7).

Understanding of the biology of plasma cell dyscrasias has been fundamental for the creation of risk scores used to identify patients at risk for progression or poor clinical outcomes, development of new drugs targeting plasma cell specific pathways, and ultimately improve the outcomes of the patients living with MM and precursor conditions. As the genomic landscape continues to be discovered, new strategies, improvements on the diagnosis, prognostication, and treatment of these patients are developed. We will review the current state and areas of interest in patients with MM and MGUS/SMM.

## Genomics in monoclonal gammopathy of uncertain significance and smoldering multiple myeloma

In contrast to patients with NDMM, who exhibit significant end organ damage and poor outcomes, a (9) subgroup of patients with a monoclonal protein, but no evidence of end-organ damage and an indolent course was identified first by Dr. Jan Waldeström. Later, Dr. Robert Kyle at Mayo Clinic demonstrated that although these patients usually are indolent, their risk of developing MM is significantly higher than the rest of the population, leading to the recognition of monoclonal gammopathy of undetermined significance. Likewise, patients with increased or marked monoclonal hypergammaglobulinemia with bone marrow plasmacytosis but with no end-organ damage, with a higher rate of progression to MM when compared to MGUS, led to the identification of smoldering multiple myeloma (SMM) as a clinical entity (10).

Given the increased risk of developing MM in these patients, clinical variables were developed to identify patients at higher risk. Two of the early and widely used clinical models in SMM were the Mayo Clinic model, which relied on the presence of ≥ 10% bone marrow plasma cells (BMPC), an M protein ≥3gr/dL, and kappa/lambda free light chain ratio ≥8 or <0.125, and the PETHEMA (Spanish) model, which included ≥95% of abnormal plasma cells on BMPC identified through immunophenotype and the presence of immunoparesis (defined as of ≥1 non-involved immunoglobulin with a >25% below lower limit of normal). High risk patients (those with 3/3 and 2/2 in the Mayo Clinic and PETHEMA models, respectively) had a median time to progression of about 24 months (11, 12). More recently, the 2/20/20 model, which relies on >2 gr/dL monoclonal protein, >20% BMPC, and an involved/uninvolved FLC ratio >20 was developed and validated in a cohort of 1996, with high-risk (2-3 factors) patients having a 2-year progression rate of 44.2% vs 6.2% in low-risk (0 factors) patients (13). Similarly, models based on immunoglobulin isotype, the presence of M protein >1.5gr/dL, and FLC ratio >8 have been used to stratify the risk of progression to MM in non-IgM MGUS (14).

Although these models are helpful to determine the risk of progression, they have several inherent limitations. Since they rely on surrogates of tumor burden, they do not indicate the actual molecular underpinnings of disease, which translate into the clinical findings in patients exhibiting indolent disease without progression despite high-risk features or patients with rapid progression despite low-risk criteria at diagnosis. Changes in hemoglobin or M-protein concentration are prognostic (15, 16), however, they are not currently adapted in any model. Recent advances have increased our understanding of the genetic events leading to myelomagenesis and have arisen as potential markers to further establish the true progressors from those with indolent disease.

### Genomics in multiple myeloma

Historically, the use of conventional cytogenetics and fluorescence *in-situ* hybridization (FISH) allowed the identification of two distinct groups of MM. One with recurrent translocations involving the *IGH* locus with another partner gene (such as *CCND1* in t(11;14) and *MMSET* in t(4;14)), and another group of patients with trisomies of whole chromosomes. One of these two alterations is almost universally present in MM, with 40-50% having IGH translocations and 50-60% of cases classified as hyperdiploid.

The time of acquisition of these alterations can be inferred based on the principle that earlier mutations will be present in a larger population of cancer cells (also known as the cancer cell fraction). Translocations and hyperdiploidy MM are clonal (CCF of >95%) and mostly mutually exclusive, suggesting they occur early during myelomagenesis and represent important driver events; this is also supported by a similar proportion of these alterations in precursor states (SMM/MGUS). However, other recurrent cytogenetic abnormalities seen in MM are subclonal and suggest a later acquisition, with each cytogenetic alteration exhibiting a variable degree of sub-clonality. For example, deletion 17p, deletion 18q, and deletion 1p were noted to be highly subclonal, whereas deletion 13q and 1q gain, are mostly clonal events suggesting they occur earlier during the malignant transformation of plasma cells (17). These cytogenetics alterations not only served as an important clue to the early events leading to MM, but also serve as the basis for the identification of patient subgroups with more aggressive phenotype and worse clinical outcomes, as it is the case of deletion 17p, *IGH*-*MMSET* (t(4;14), and 1q amplification (18, 19).

**Figure 1:**
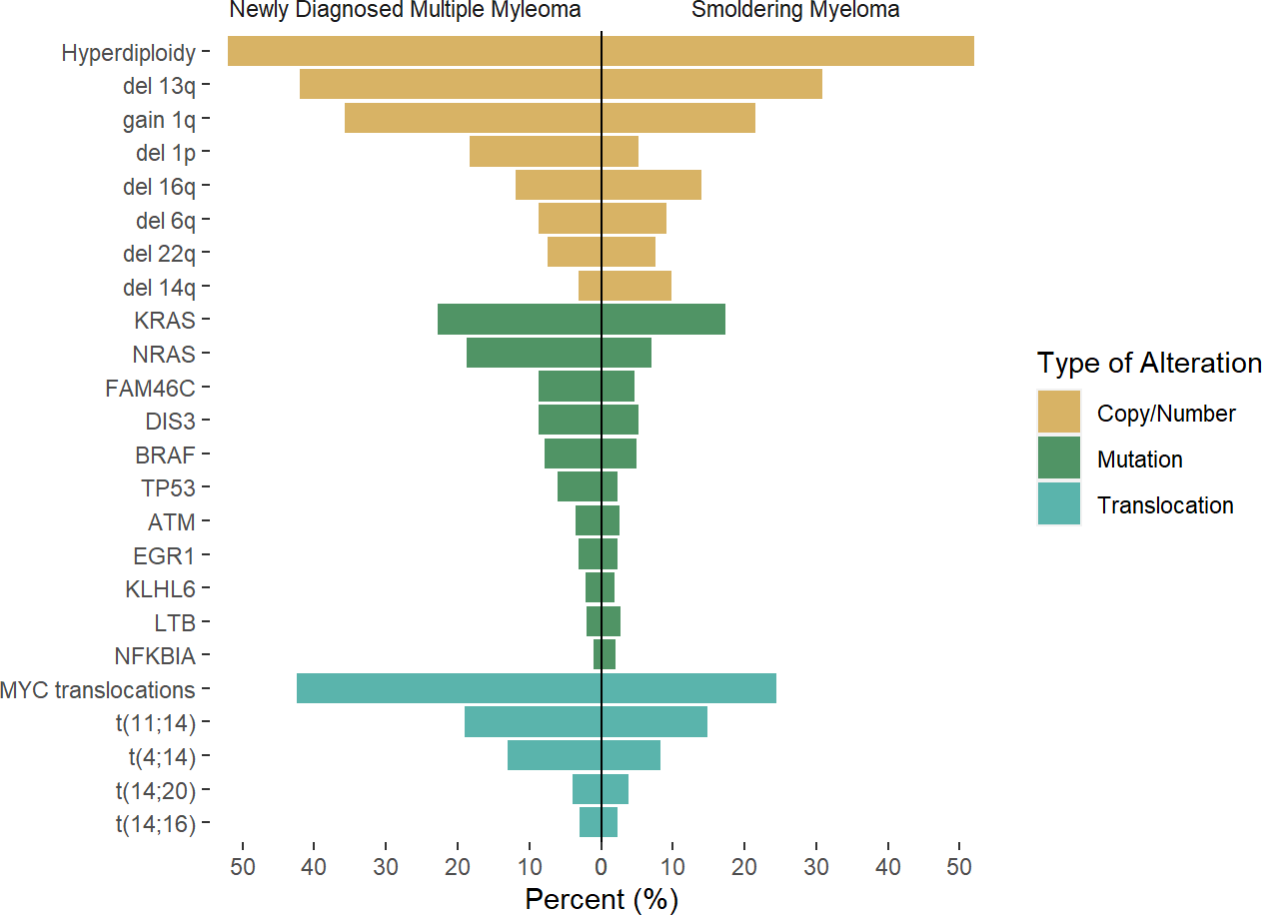
Rate of genetic alterations across newly diagnosed multiple myeloma and smoldering myeloma. Results of previous reports by Boyle et al (22), Bustoros et al (17), Misund et al (23), Walker et al (24) were pooled to the described average rate of the described alteration, as there is variability in likely secondary to differences in methodology used. Differences across studies and methodology used may account for differences.

The introduction of next-generation sequencing and the increasing availability of these platforms has led to a new understanding of the characteristics of MGUS/SMM and the evolution towards overt MM. Whole-exome sequencing (WES) allowed the identification of several mutations in MM, with the most common pathways involved the MAPK pathway (with mutations in the *KRAS*, *NRAS*, and *BRAF* genes present in roughly 40% of cases), DNA damage response (including *TP53* and *ATM*), and the NF-kB response pathway (20, 21) **Figure 1**. Unlike acute myeloid leukemia in which point mutations are main driver events and define a clinical phenotype, mutations in MM are often subclonal (later events) (25) and are largely variable from patient to patient, with only a small fraction of patients sharing common mutations, However, there is evidence some of the most common mutations described above are pathogenic and have clinical impact, as they are associated with certain cytogenetic subtypes as is the case for *DIS3* and t(4;14), and have been associated with worse clinical outcomes as is the case for *TP53* and *ATM* mutations (21), whereas many of the less frequent mutations encountered in few patients do no have particular associattion with disease subtype or cytogenetic group, suggesting they are rather “passenger” events that occur as a result of subclonal evolution.

Although WES was widely used initially in MM, one of the limitations is missing structural variants (SV’s) that may also play an important role. Large SV’s events have been widely described in both hematology and solid malignancies and are associated with chromosomal events leading to deletion, translocation, and tandem duplication causing known pathologic copy number/number or enhancer hijacking in oncogenes and tumor suppressor genes (26, 27). In the case of MM, these events were initially considered to be rare, however, the use of long-read whole-genome sequencing (WGS) platforms have allowed the identification of 1 or more complex SV’s in 68% of cases (28). Chromotrypsis, chromoplexy, and templated insertions were present, in 38%, 11%, and 19% of newly diagnosed patients, and have been associated with worse clinical outcomes in addition to close association with certain disease subgroups, as is the case for t(11;14). Furthermore, novel hotspots in previously considered non-coding regions have been identified, and have been linked to complex SV’s associated with other clinically relevant alterations such as 17p deletion and 1q gain (28, 29). Importantly, these complex SV’s have been associated with simultaneous acquisition of >1 driver event, often in unrelated regions that would otherwise be considered as independent events.

### Myelomagenesis: Genomic basis for precursor development and progression to multiple myeloma

After the initial description of the genetic landscape in MM was described and expanded in multiple studies (21, 24), a similar approach was taken for MGUS/SMM. As mentioned before, patients with SMM have a similar prevalence of recurrent translocations and hyperdiploidy as those seen in MM, highlighting the clonal nature of these events.

Recent efforts to identify the mutational landscape in SMM have shown very similar results to those seen in NDMM. A cohort of 214 patients with SMM who underwent WES mutations in the MAPK pathway (*KRAS*, *NRAS*, and *BRAF*) was the most common finding, seen in 48% of patients, followed by mutations involving DNA repair pathways (*TP53*, *ATM*, and deletion 17p, 21% of cases), and NF-kB pathway (10% of cases), while other mutations such as *DIS3, FAMC46C, ZNF292, RB1, CDKN2C* were present in less than 10% of cases. Again, some of these mutations are highly subclonal, suggesting the acquisition of these events occurred at a later stage than foundational events, but often present to provide fitness to the tumor cells. Based on these findings, the mutations in the MAPK pathway, DNA repair, and *MYC* alterations were identified as risk factors for progression, and when added to the 2/20/20 model, they increased the performance when compared to the model based on clinical variables (17). The importance of these alterations in the progression of SMM was confirmed as well in additional studies (22, 23), also highlighting the higher prevalence of *MYC* alterations in progressor SMM and NDMM (**Figure 1**).

The role of SVs in the progression of precursor conditions was evaluated in a cohort of 18 MGUS and 14 SMM and compared to 80 MM. Cases with “stable” MGUS/SMM had a lower SV’s burden overall. This difference was even more striking for complex SV’s, with only 1 stable case having evidence of chromothripsis vs 47% and 41% of progressor precursors having evidence of chromothripsis and templated insertions, respectively, and with a similar burden of SV’s when compared to established MM cases (30). Similar findings were seen for copy number alterations, with a higher rate of MM recurrent chromosomal events such as gain 1q, deletion 8p, deletion 6q in cases of progressor precursors, despite no significant difference in aneuploidies or recurrent translocation between these two groups.

Mutational signatures analysis can inform on the mechanism leading to mutations and SV’s. In the case of MM, several mutational signatures have been identified and seem to be relatively constant across cases. These include evidence of activity of the enzyme activation-induced cytidine deaminase (AID) across immunoglobulin locus (known as canonical AID, c-AID) and in non-immunoglobulin areas (known as non-canonical AID, nc-AID), APOBEC, “clock-like” signatures (related to constant mutation over time (31)). Patients with progressor precursors have been associated with higher evidence on nc-AID and APOBEC when compared to stable precursors, suggesting differences in underlying mutational events in both subgroups. In addition, the overall mutational rate for progressor conditions mirrors the one seen in NDMM (17, 30). In addition, in matched samples of a patient with progressive SMM, most cases (>70%) had preserved subclonal structure with no new subclones and no significant subclonal expansion, suggesting the necessary events for malignant transformation are already present at the SMM/MGUS stage, and that subclonal evolution may be a marker of higher risk but not necessarily causal of progression, following a model of static evolution, with a minority of cases exhibiting acquisition of new lesions and/or changes in subclone structure (17, 22, 30).

**Figure 2:**
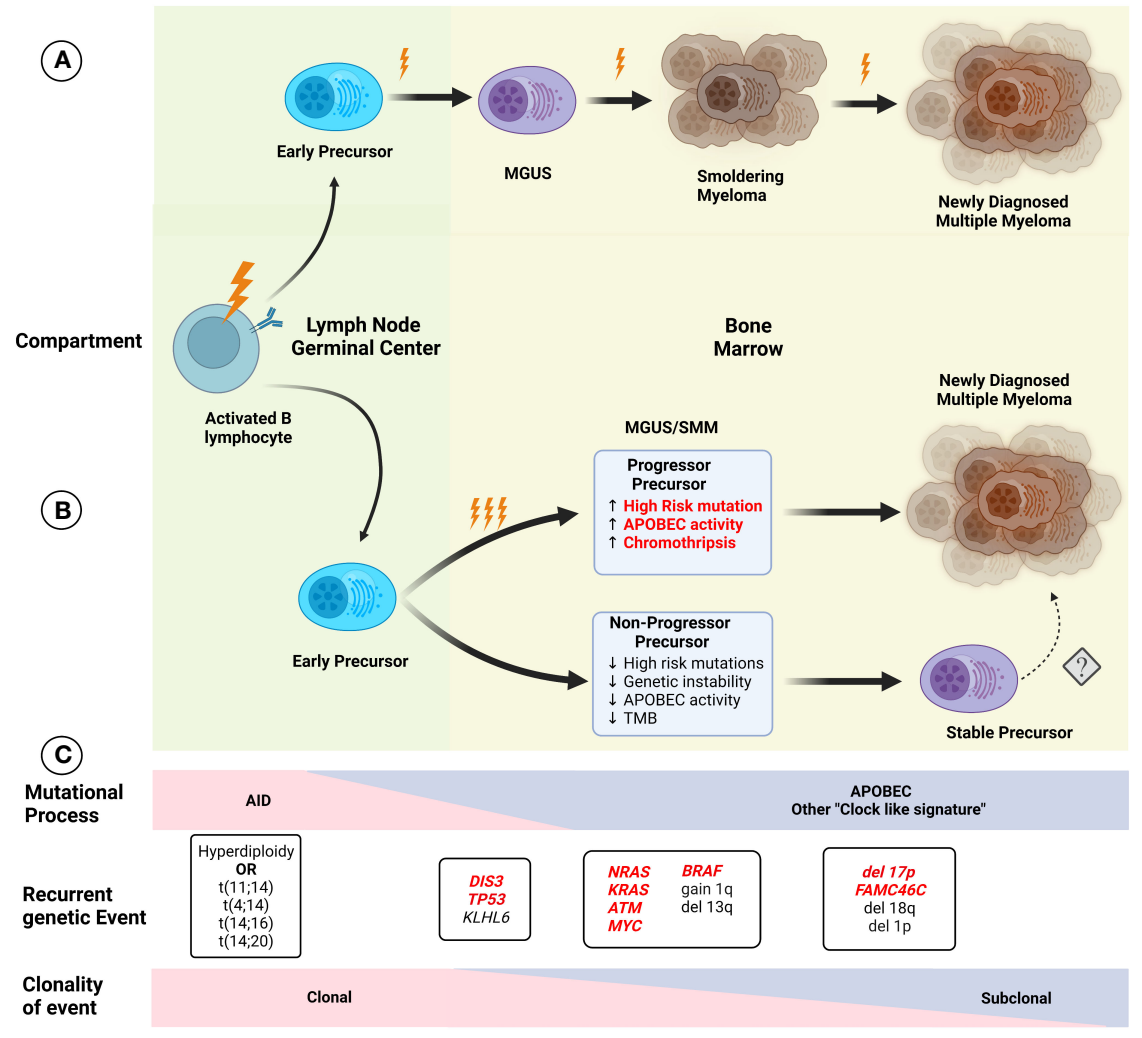
Initial models of **(A)** myelomagenesis postulated the appearance of a precursor clone which required acquisition of mutations and/or chromosomal abnormalities in a stepwise fashion leading to the observe progression from monoclonal gammopathy of uncertain significance (MGUS), to smoldering myeloma (SMM) and finally multiple myeloma (MM) after enough pathogenic event have occurred. **(B)** Based on recent findings, the paradigm of myelomagenesis has shifted, with highly clonal events, such as recurrent translocations involving *IGH* gene and hyperdiploidy representing fundational events. The role of AID initially, and later APOBEC as important mutational process likely contribute to acquisition of additional events (and possibly several during one event, described as “catastrophic evolution”). At the moment of clinically evident precursors (MGUS/SMM), two groups of are present, one characterized by higher genomic instability, with higher tumor mutation burden (TMB), increased chromoplexis and other complex structural events, and increased in pathogenic events, whereas the “non-progressor” precursor have low risk of progression over time and lack some of the features described before, although the potential risk of conversion to a more aggressive disease with progression to MM is not clear (denoted by dashed arrow). Several characteristics in the precursor clone (highlighted in red) indicate a higher risk of progression and potentially could be used clinically for risk stratification. **(C)** The effect of different mutational process potentially varies over time, with an increased in AID, while APOBEC and other process such as “clock-like” signatures are present at a later time. Except for early foundational events described before, other alterations display different degrees of sub clonality, with most cases of MM exhibiting relatively preserved clonal structure upon progression.

Improvement in the understanding of the genetic events present in NDMM has led to changes in the established model of myelomagenesis. Initially, MM development was considered to follow a step-wise model, such as colon cancer, where specific mutations occurred over time leading to precursor conditions and eventual progression to malignancy. However, this model seems to not be representative of MGUS/SMM and MM, especially given the relatively preserved subclonal composition of most patients with progressive precursors when compared to matched NDMM samples. Early events leading to hyperdiploidy and translocations involving the *IGH* locus seem to be related to exposure to AID in the germinal center, while APOBEC and “clock-like” signatures contribute to later events, presumably once migration to the bone marrow has been completed (**Figure 2**). This conceptual framework is not only relevant to understanding the events leading to myelomagenesis but also provides the basis for the identification of potential progressor vs stable precursors clinically, potentially improving our stratification of these patients. Additionally, other cytogenetic alterations such as deletion 17p, 1q gain, t(4;14), t(14;16), and 13q deletion/13 monosomy have also been associated with an increased risk of progression to MM, leading to the addition of these findings to the previously described 2/20/20 model (13). The frequent co-occurrence of copy number abnormalities, recurrent translocations, and mutations led to the identification of well-defined molecular subgroups of NDMM and RRMM (32, 33), which not only differ by expression of certain markers (such as CD20 enrichment in CD-1, associated with t(11;14), but also exhibit differences in clinical phenotype and prognosis. Recently, using a set of 42 alterations (single nucleotide variants, translocations, and copy number alterations), 6 well-defined molecular subtypes of SMM were identified, which not only had significant overlap in terms of co-existing abnormalities with previously defined MM genomic subgroups but were also prognostic in terms of TTP to active MM. Furthermore, the use of these genomic classifications coupled with the 20/2/20 model improved the prediction performance of the model. In addition, the presence of high-risk subgroups identified patients at higher risk of progression within the clinically defined high and intermediate risk (34).

Additionally, recent insights on additional high-risk alterations in NDMM, suggest that not all chromosomal cytogenetic alterations confer the same degree of risk. Biallelic *TP53* loss (either through deletion or mutation) was associated poor overall survival when compared to patients with no or only 1 *TP53* event, suggesting biallelic events are relevant during clonal evolution and potentially more informative than isolated, mono-allelic alteration currently being captured (35). The importance of 1q copy number gains as a prognostic factor in NDMM has also been highlighted recently as these patients have poor outcomes and mirror those considered to have high risk cytogenetic features such as del 17p and t(4;14). In addition, the “burden” of 1q gain seems to be relevant, as patient with 1q amplification (>3 copies) have worse outcomes when compared to patients with 1q gain (3 copies) (18, 36). Proteosome inhibitors (PI’s) are thought to abrogate the effect of high risk cytogenetic features (del 17p and t(4;14)) on outcomes, however, 1q amplification may indicate therapeutic resistance, as patients with 1q amplification enrolled in the FORTE trial had poor outcomes despite upfront Carfilzomib use, whereas those with del 17p and t(4;14) had similar outcomes to those with standard risk cytogenetic (37, 38).

Limited panels detecting known clinically relevant mutations and fusions have been already developed and are widely used in other disease settings, such as acute myeloid leukemia (39). These panels could identify prognostic mutations in MAPK pathway, DNA repair, *MYC*, and other potential genes. The identification of specific genomic signatures, tumor mutational burden, and complex SV’s, although associated with higher rates of progression, has the limitation of requiring much broader coverage of the genome, potentially limiting the current clinical application of these markers. However, high-throughput next-generation platforms are evolving quickly with increasing efficiency and decreasing cost per patient, potentially opening the doors for a future wide-scale implementation of this technology in clinical practice.

## Future biomarkers in SMM and multiple myeloma

The use of circulating tumor DNA (ctDNA) has the potential to identify clinically relevant mutations, and indicate response or residual disease without the need for an invasive procedure, hence the term “liquid biopsy”. It has been applied for several solid and hematologic malignancies and is widely used in clinical practice (40). In the case of MM, ctDNA burden correlates with higher 70-GEP and prognosis, with patients with higher burden having worse PFS and OS (41). Furthermore, it has been proposed as a tool to detect treatment failure and subsequent relapse in the RRMM setting (42). As mentioned before, the integration of SNV’s, translocation, and copy number changes into defined molecular subgroups of SMM improve the performance of 2/20/20 model (34), but will lack of widespread access to this technology remains a limitation.

The availability of a minimally invasive test that could inform on disease stratification and potentially provide information on progression would be of great use in SMM. However, some limitations exist for ctDNA in the case of MGUS/SMM. First, patients with precursor conditions tend to have much lower ctDNA, possibly related to a lower tumor burden. Second, there is a lack of correlation between the findings in the bone marrow and ctDNA, with the latter missing recurrent translocation and other point mutations, as described by Deshpande et al. in a cohort of 25 SMM patients with 14 patients having translocations in the bone marrow and none found in ctDNA. Similarly, there was only matching of mutations in 4/13 patients (41), suggesting ctDNA may be limited either by DNA quantity or lack of correlation. Upon follow up, 2/25 patients had an increasing burden of ctDNA and expansion or appearance of new mutations, however, only 1 of these patients progressed to MM.

More promising is the use of circulating tumor cells (CTC) as a marker for high risk in precursor conditions. CTC’s are frequently found in NDMM, with some cohorts describing up to 95% of NDMM cases having detectable CTC using highly sensitive methods such as next generation flow cytometry (43). Additionally, higher burden of CTC’s correlates with higher ISS stage in NDMM (43), and worse outcomes in NDMM and RRMM, representing an atractive biomarker (44, 45). Furthermore, differences in RNA expression profile of CTC’s when compared to bone marrow or extramedullary (EMD) matched samples demostrate the complex and dynamic process of malignant plasma cell egress and potentially serve as the basis for markers to identify patients at high risk of developing EMD disease (46). CTC’s can be detected in SMM, with up to 78% of patients having detectable CTC’s using next generation flow cytometry (43), and higher burden of CTC’s are associated with an increased risk of of progression (43, 47), however, the treshold to define this high risk population has not been well defined and likely will vary depending on the type of assay used, however, in a recent cohort of 230 SMM with detectable CTC’s, patients with ≥ 0.02% CTC’s had a median TTP of 11 months (43), which is similar to what was known as “ultra-high risk” SMM, and were later classified as NDMM after the 2014 updated IMWG diagnostic criteria (48). Similarly, higher rates of CTC’s correlated with MGUS progression, again highlighting the potential role of this marker in evaluating precursor conditions (49).

The need for refinement of existing ctDNA assay or development of new technologies is needed in this population, as the use of ctDNA in precursor conditions seems to be limited at this point. Some of the limitations of CTC’s include the lack of widespread access to next generation flow cytometry, and lack of standarization of optimal tresholds to indentify high and low risk subgroups have varied, as prior results have varied likely secondary to differences in methdology and patient selection. However, both CTC’s and ctDNA represents attractive areas for research as they represent minimally invasive methodologies which could allow sequential sampling allowing for longitudinal follow up of patients. Additionally, they do not have the sampling bias that is seen with bone marrow samples, potentially allowing to capture the heterogeneity of MM.

## Genomics studies provide insights into resistance mechanism to MM therapies

Advances in MM therapy have come about due to therapies that target vulnerabilities of the plasma cell such as high protein load (proteasome inhibitor (PI); bortezomib, ixazomib and carfilzomib), dependence on specific transcription factors such as IKZF1 and IKZF3 which are degraded by immunomodulatory drugs (IMiDs; thalidomide, lenalidomide, and pomalidomide), the susceptibility of B cells to glucocorticoids (Dexamethasone) and the presence of specific B cell markers that can serve as targets for monoclonal antibodies and CAR-T cells (BCMA). Survival of patients with MM has significantly improved over the past decade with the introduction of these therapeutic strategies (5). However, these therapies are not curative, and nearly most of the patients with MM eventually relapse and require further therapy. In this part, we will discuss the genetic aberrations related to resistance to various therapies in MM (**Table 1**).

**Table 1:** Proposed mechanism of resistance and possible.

Target	Drugs	Mechanism of Resistance	Potential Methods
Proteasome Inhibitor (PI)	•Bortezomib •Carfilzomib •Ixazomib	•Downregulation of Ire1-Xbp1 pathway. •Mutations in *XBP1* and in genes of the Proteasome-complex (such as *PSMB5*). •Overexpression of Proteasome-complex proteins subunits, overexpression. •Overexpression or mutations of genes involved in Proteasome-complex conformation such as Chaperones (*PSMG2*)	•Development of new PI with increased affinity or lack of cross-resistance.
Immunomodulatory Drugs	•Thalidomide •Lenalidomide •Ixazomib	•Downregulation of CRBN-complex proteins •Mutations in *CRBN* and other genes part of the CRBN-complex •Upregulation of other pathways such as MAPK/ERK and MYC •Mutations in other genes such as *TP53*, *IKZF3*, and other associated pathways.	•Novel compounds with increased affinity or targeting alternative components of the CRBN-complex.
Anti-CD38 Antibodies	•Daratumumab •Isatuximab	•Downregulation of CD38 •Upregulation of complement inhibitory proteins (e.g.; CD55, CD59) •Upregulation of IL-6-JAK-STAT3 signaling pathway or other antiapoptotic pathways	•Upregulation of CD38 expression with alternative approaches. •Enhancement of complement mediated cytotoxicity.
BCMA Directed Therapy	•Belantamab Mafodotin •CAR-T •Bi-especific T-cell engager	•Homozygous deletion and mutations in *TNFRSF17* encoding BCMA. •Increased cleavage of BCMA secondary to γ-secretase activity leading to increased sBCMA	•Use of γ-secretase inhibitors. •Optimizing BCMA targeting by monoclonal antibodies and CAR-T cell constructs.
Bcl2-Inhibitors	•Venetoclax	•Downregulation of *BIM* and upregulation of *BCLXL, AURKA, BIRC3*, and *IL-32.* •Upregulation of Bcl-2 protein, MAPK/ERK and PI3K pathways. • Unclear role of point mutations in *BCL2* in resistance.	• Synergistic use of other compounds such as PI to enhance Venetoclax sensitivity.
XPO1 inhibitor	•Selinexor	•Overexpression of E2F1 leading to increased E2F1 localization in nucleus	• Requires further validation

## Proteasome inhibitors

Proteasome inhibitors (PIs) were developed for the treatment of MM, and it has dramatically improved survival and treatment responses (50). These molecules driven apoptosis are related to the role of the ubiquitin proteasome pathway (UPP) in protein turnover. The UPP degrades intracellular aberrant or unnecessary proteins (misfolded and potentially toxic proteins) in eukaryotic cells. This process is essential to maintain cellular homeostasis and UPP is involved in apoptosis, cell survival and cell cycle control (51). It includes different steps: polyubiquitylation, deubiquitylation, and degradation of the target protein. The polyubiquitylation is mediated by ubiquitin activating enzyme 1 (E1), multiple ubiquitin-conjugating enzymes (E2) and ubiquitin-protein ligases (E3). The deubiquitylation is mediated by the proteasome regulatory subunits, which include both the 19S particle in the constitutive proteasome and the 11S particle in the immunoproteasome. The proteasome degrades the proteins *via* the function of the 20S core particle catalytic sites, and release oligopeptides. The catalytic sites of the 20S core particle have chymotrypsin-like (β5), trypsin-like (β2), and post-glutamyl peptide hydrolyzing, or caspase-like, (β1) activities (50).

Three proteasome inhibitors (Bortezomib, Carfilzomib and Ixazomib) have been approved by the US Food and Drug Administration (FDA) for treatment of MM. These molecules are administered subcutaneously, intravenously and orally, respectively and inhibit the 20S proteolytic core of the proteasome with different degree of inhibition of β5/chymotrypsin-like, the β2/trypsin-like or β1/post-glutamyl peptide hydrolyzing activity.

### Mechanism of resistance to proteasome inhibitors

Proteasome inhibitors dramatically improved survival in myeloma patients; however, relapses are frequent and acquired resistance to treatment eventually emerges. The exacerbation of ER stress-related cytotoxicity induced by PIs could be reversed by the downregulation of Ire1-Xbp1 pathway. When stimulated by ER stress, Ire1 (one of three ER transmembrane proteins) transduces stress signals to the nucleus and activates the UPR *via* the activation of a transcription factor, Xbp1. Two *XBP1* mutations have been detected in PI refractory patients. It was also reported that Xbp1s-negative tumor cells are resistant to PI; The mechanism of this resistance was explained by the decommitment to plasma cell maturation, and the lower IgG secretion in Xbp1-negative cells in comparison to PI-sensitive cells, as plasma cells depend on Xbp1s for Ig synthesis (52). Furthermore, bortezomib resistance was associated with mutations in *PSMB5*, gene encoding proteasome subunit β5, in myeloma cell lines (53). These mutations affect the PI-binding pocket S1 leading to conformational and steric changes to the proteasome drug- binding. However, somatic mutations of *PSMB5* were rarely detected in patients and Carfilzomib response was less affected by *PSMB5* mutations, due to its unique structure and binding (54). Due to their different targets and mode of action, MM patients who relapse on bortezomib are responders to treatment with Carfilzomib. Carfilzomib targets the 20S subunit and chaperones and stress-response regulators (55). Another mutation in proteasome assembly chaperone 2, *PSMG2* gene has been also detected in one patient refractory to bortezomib (56). This protein is involved in mammalian 20S proteasome maturation, and exonic deletion of *PSMG2* has also been reported in MM (57). Mutations in *XBP1* (p.L314Ffs) was found in one patient resistant to PI at the time of tumor sampling (58). However, these potential findings need to be verified in larger studies to determine the scale and effect of such mutations in response to PIs.

Other mechanisms of PI resistance include overexpression of the proteasome subunit β5, overexpression of other subunits such as β2 and β1, downregulation of 19S proteasomal subunits in MM cell lines and MM patients (55), or overexpression of microenvironmental proteins (IL-6 and IGF-1) and chaperones (59, 60). Very recently, Li et al. showed that a deubiquitylase USP12 (Ubiquitin specific protease-12) is involved in bortezomib resistance in myeloma cells (61). In addition, proteins involved in proteasome function, oxidative stress, defense response and regulation of apoptotic process could be potential biomarkers of resistance to PIs (62).

## Immunomodulatory agents

Immunomodulatory agents are cornerstone in MM therapy. The first clinical trial assessing the effects of thalidomide, an immunomodulatory drug (IMiD), in 1999 showed that it is active against advanced myeloma in patients who had failed stem cell transplantation (63). Similar clinical trial was then conducted with 16 patients with relapsed myeloma and confirmed the previous results (64). An analogue or derivative of thalidomide (now known as lenalidomide) was tested in 2002 and showed good response in relapsed MM patients without the neurotoxicity and other adverse effects described after thalidomide treatment (somnolence, constipation, and neuropathy) (65). This drug increased TNF-alpha production by both CD4+ and CD8+ T cells (66). Another study showed that IMiD overcomes CTLA-4-Ig inhibitory effects of T cell proliferation (67). lenalidomide has been used in combination with dexamethasone for the treatment of adult patients with MM. It has also been used in combination with proteasome inhibitors. IMiDs not only target myeloma cells but also exert an indirect effect by activating the proliferation of the cytotoxic T and NK cells (65).The mechanism of IMiDs action was clearly described in studies, elucidating that cereblon (CRBN) plays an important role in mediating IMiDs anti-tumor effects (68). CRBN is a ubiquitously expressed E3 ligase protein, and it is the primary target of IMiDs (69). CRBN is part of a functional E3 ubiquitin ligase complex together with the DNA damage-binding protein-1 (DDB1), Cullin 4A and Roc1 (CUL4-RBX1-DDB1-CRBN) and acts as a substrate receptor for client proteins to be ubiquitinated and degraded by proteasome. IMiDs binding to cereblon E3 ligase complex induces the ubiquitination and degradation of transcription factors Ikaros and Aiolos, and subsequently downregulates IRF4 and c-MYC (70–72). These factors are essential for myeloma cells proliferation and survival. Furthermore, Ikaros and Aiolos are repressors of IL-2 (73), leading to T cell expansion in the tumor microenvironment.

### Mechanism of resistance to Immunomodulatory drugs

In human MM cells, CRBN down-regulation resulted in the development of IMiD resistance (72, 74, 75). In a xenograft model, resistance to pomalidomide plus dexamethasone, but not lenalidomide plus dexamethasone was related to decreased CRBN expression but the resistance to both combinations is accompanied by upregulation of MEK/ERK pathway; this was confirmed by the inhibition of ERK which sensitizes resistant cells (76). In patient samples treated and became refractory to lenalidomide, a decrease in CRBN and increase in MYC expression were observed (77, 78).. In another study, higher CRBN expression level was associated with better PFS in patients treated with pomalidomide + low-dose dexamethasone (79). CRBN downregulation could be related to epigenetic modifications as epigenetic therapy re-sensitized IMiDs-resistant cell lines and changed the global chromatin accessibility associated with IMiDs resistance (75).

**Figure 3:**
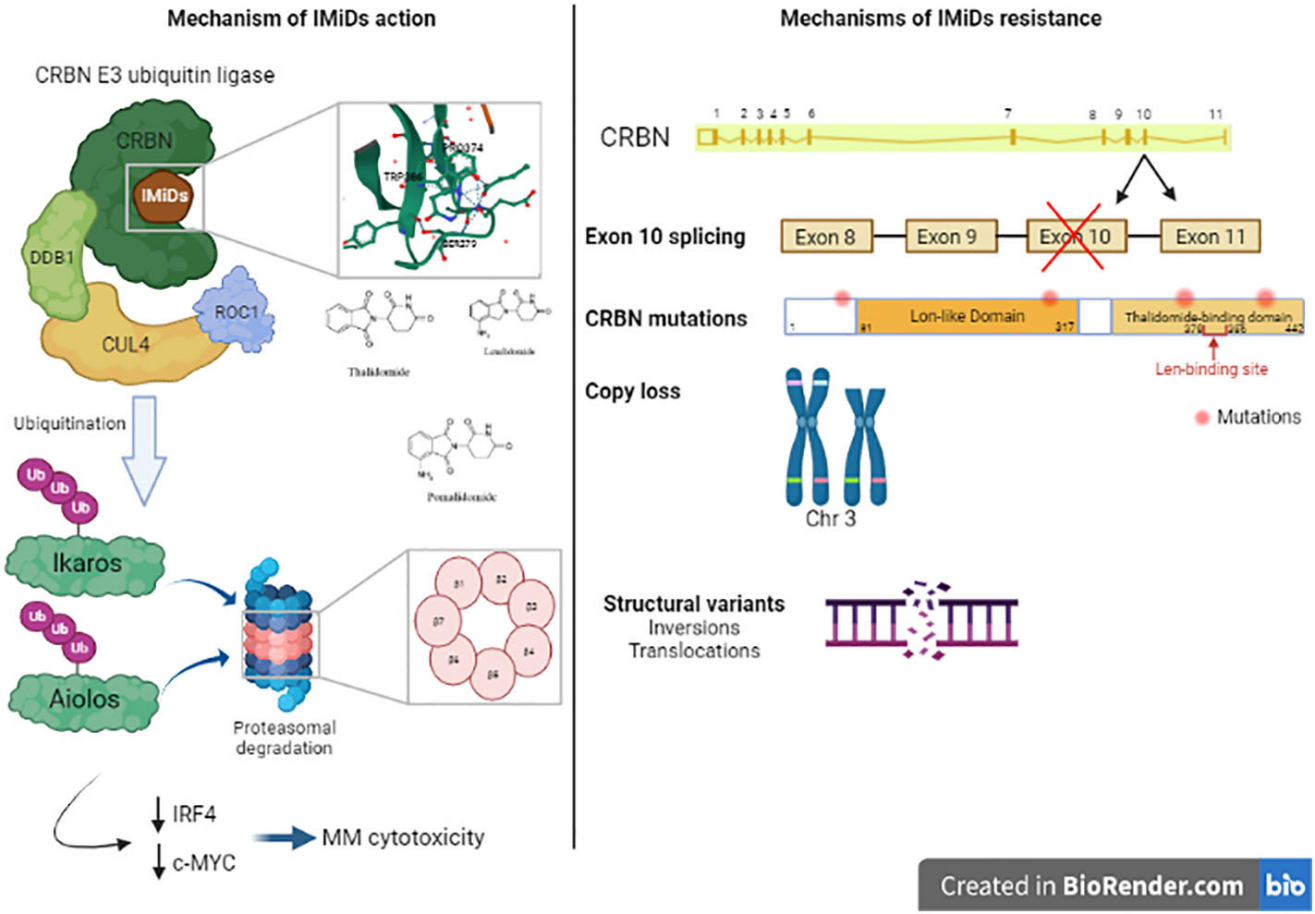
Mechanism of action of Immunomodulatory drugs (IMiDs): Immunomodulatroy drugs interacts with Cereblon (CRBN), which is a part of CRBN E3 ubiquitin ligase and induces the ubiquitination of Ikaros and Aiolos and their degradation which decreases the expression of IRF4 and c-MYC, two important factors in MM cell’s survival. This leads to MM cytotoxicity. Mechanisms of IMiDs resistance: Acquired resistance to IMiDs is associated with different genetic alterations in CRBN gene including exon 10 splicing, CRBN gene mutations, copy loss or structural variants like inversions or translocations in CRBN locus. These alterations impair CRBN function and consequently, MM cell’s response to IMiDs.

Recently, using a MM gene panel, Kortüm et al. showed an increased prevalence of mutations in CRBN and CRBN pathway genes (*CRBN*, *CULB4*, *IRF4*, *IKZF1*) in IMiDs (lenalidomide) refractory patients compared with newly diagnosed MM patients (58). These mutations in *CRBN* were either located within the IMiD-binding domain or occurred at sites truncating the protein. These mutations were not detected at the earlier time point when the patients were sensitive to IMiDs. *In vitro*, these mutations introduced in OCI-MY5 cell line caused a lack of response to lenalidomide. Another study showed other mutations in CRBN in MM patient who was initially responsive to thalidomide and lenalidomide treatment, who acquired resistance to lenalidomide over the disease course, and who was finally unresponsive to pomalidomide treatment at the time of tumor sampling. In this patient, a point mutation (p.Arg283Lys) and a truncating mutation (p.Glu99X) were detected within CRBN gene (56). Suggesting an association to IMiD resistance, another CRBN mutation has been identified in a patient unresponsive to initial lenalidomide and later pomalidomide treatment, this mutation is located in close proximity to the IMiD-binding site of the gene (58). A specific *CRBN* mutation (p.Cys326Gly) has also been detected at relapse in a patient treated with lenalidomide showing that this mutation contributed to lenalidomide resistance and clinical relapse (80). The cysteine amino acid caused by this mutation is involved in the Zinc finger motif, leading to protein misfolding and aggregation which destabilizes the IMiD binding domain of the protein (81). In a larger cohort of 198 newly diagnosed (ND) patients, 203 lenalidomide-refractory and 54 pomalidomide-refractory patients, WGS revealed that *CRBN* mutations occurred in 2.2% of lenalidomide-refractory and 9% of pomalidomide-refractory patients and 0.5% in ND-patients (82). Furthermore, resistance to IMiDs is also due to other genetic aberrations in CRBN such as copy number loss and structural variations (translocation or inversion) (82). Additionally, exon 10 splicing or the deletion of pomalidomide/lenalidomide binding domain is also associated with IMiDs resistance. The ratio of exon 10 spliced/full-length CRBN transcripts was increased in pomalidomide-refractory compared to ND or lenalidomide-refractory patients affects lenalidomide response in myeloma cell lines (82, 83). The presence of these CRBN aberrations were associated with significantly reduced PFS and OS in lenalidomide-refractory MM (82). Other mutations were detected in genes coding for the core CRL4CRBN E3-ligase complex and COP9 signalosome (80). There are 42 genes termed “CRBN/IMiD genes”, and 12/42 genes showed mutations in a dataset of 56 patients from the UK National Cancer Research Institute Myeloma XI trial at presentation, relapse or at both time points (80). These mutations are associated with relapse after lenalidomide treatment since 9/17 mutations arose in patients who had received lenalidomide and 6/9 had a higher cancer clonal fraction (CCF) in the relapse sample, suggesting that they may have been selected for by exposure to treatment. Furthermore, mutations in other related genes (*TP53* and *IKZF3*) have been also detected in patients’ samples after lenalidomide treatment (77). An overview of the mechanisms of resistance to IMiD’s are described in **Figure 3**.

## CD38 and BCMA directed therapies

The development of immunotherapies in the treatment of MM has emerged with the use of monoclonal antibodies anti-CD38 (daratumumab (DARA) and Isatuximab) and the development of Anti-BCMA CAR-T cells and bispecific antibodies.

DARA, a human CD38-specific IgG1 antibody, was first approved in 2015 as a single agent therapy in RRMM patients (84). CD38 is a transmembrane glycoprotein expressed on MM cells and at low levels on normal lymphoid and myeloid cells (85). CD38 has ectoenzymatic activity in the catabolism of extracellular nucleotides and adhesion function related to its interaction with CD31 or hyaluronic acid which regulates the cell’s migration. CD38-targeting antibodies kill MM tumor cells *via* Fc-dependent immune effector mechanisms including complement-dependent cytotoxicity (CDC), antibody-dependent cell-mediated cytotoxicity (ADCC), antibody-dependent cellular phagocytosis (ADCP), and apoptosis upon secondary cross-linking (86). These mechanisms are dependent on the interaction of the Fc region of the antibody with Fcγ receptors (FcγRs) expressed on immune effector cells.

DARA combination with IMiDs (lenalidomide) and PIs (bortezomib) improved anti-MM activity and led to significant improvements in clinical outcomes in RRMM patients (87, 88) or untreated myeloma (89, 90), and DARA combinations were approved by FDA for the treatment of MM patients with at least one prior line of therapy in 2016. Moreover, further analysis of these trials showed an improvement of outcome of high-risk patients (del17p, t(4:14) or t(14:16)) in comparison to Rd and Vd only (91, 92).

DARA efficacy is correlated to the CD38 expression on MM cells (93). Several case reports showed CD38 downregulation in MM patient relapsing after DARA treatment as an escape mechanism (94, 95). In a subset of 102 patients treated with DARA monotherapy, CD38 pretreatment levels on MM cells were significantly higher in responders to DARA vs. non-responders (96). This study suggested the implication of CD38 downregulation and complement-inhibitory proteins CD55 and CD59 upregulation in the resistance to daratumumab. Another study from the same group suggested the implication of trogocytosis, cell membrane transfer from MM cells to monocytes and granulocytes, in the CD38 downregulation on the MM cell surface (97). CD38 downregulation was observed during DARA treatment and at the time of progression but CD38 expression increase after stopping DARA (96). Preclinical studies showed that all-trans retinoic acid (ATRA) increased CD38 expression and reduces CD55 and CD59 expression on MM cells and reverts DARA-resistance *ex vivo* (96, 98). However, ATRA-combined treatment did not enhance daratumumab response in MM patients, this may be explained by the transient upregulation of CD38 expression (99). In addition, HDAC inhibitor, Panobinostat also induces CD38 upregulation on MM cells *ex vivo*, leading to increased antimyeloma efficacy of daratumumab through an increase in ADCC (100). CD38 downregulation and reduced DARA-mediated antibody-dependent cellular cytotoxicity are observed in presence of bone marrow stromal cells (BMSCs) supernatant and IL-6 *via* JAK-STAT3 signaling, suggesting that bone marrow microenvironment could have an important role in protecting myeloma cells from DARA-induced cytotoxicity (101). In addition, BMSCs confer protection of MM cells against Dara-induced ADCC; this resistance is possibly related to the upregulation of anti-apoptotic proteins survivin and Mcl-1 (102).

BCMA (B-cell maturation antigen) is another ideal target for MM immunotherapy, it is expressed on MM cell lines and malignant plasma cells with high prevalence and its expression increases during disease progression from MGUS to SMM to MM (103–105). BCMA is involved in tumor proliferation *via* the delivery of pro-survival signals in MM cells and is ubiquitously expressed on the surface of MM cells. Anti-BCMA Chimeric Antigen Receptor (CAR) T cell therapy has been developed in MM and improved outcome in poor prognosis population like triple-refracted patients. The KarMMa study assessed the efficacy of idecabtagene vicleucel (ide-cel), a BCMA-directed CAR T cell therapy, in 128 refractory MM patients (≥3 prior regimens). This study showed that 76% of patients achieved CR and 29% MRD-negative status maintained for up to 12 months (in 60% of MRD-negative patients) (106). However, some patients relapsed following this CAR-T cell therapy. Da Vià et al. identified a homozygous deletion of BCMA-encoding gene (*TNFRSF17*) as a mechanism of escape from CAR-T cell therapy in relapsed patient (107). They observed a heterozygous deletion of this gene in MM patients even before treatment which can be a predicting marker for the BCMA-targeting therapies. Furthermore, another group showed that deletion of 16p, including the BCMA locus, and a truncating mutation in BCMA gene occur after anti-BCMA CAR-T cell therapy causing lack of BCMA expression at the time of relapse and explaining the lack of response to second CAR T cell infusion (108). The efficacy of BCMA-targeting therapies could be also dependent on the soluble BCMA (sBCMA) levels. Soluble BCMA release in the serum is due to the γ-secretase-induced shedding, a ubiquitous intramembranous protease that sheds membrane bound BCMA (109). sBCMA can bind and interfere with anti-BCMA antibodies, thus, drugs inhibiting gamma-secretase could enhance the efficacy of BCMA antibodies by reducing shedding of BCMA form the cell surface and subsequent interference of BCMA-specific antibodies by sBCMA (110). Soluble BCMA is also a biomarker for myeloma prognosis (111). The level of serum sBCMA is correlated with bone marrow plasma cell levels (112). sBCMA levels were higher in MGUS, and SMM patients who progressed to active MM showing that sBCMA may be a useful prognostic biomarker in MM (113).

## Venetoclax sensitivity in MM

Venetoclax (ABT-199) is a selective antiapoptotic protein B-cell lymphoma 2 inhibitor that induces apoptosis by displacing proapoptotic proteins from the antiapoptotic protein Bcl-2 (114). Venetoclax efficacy in inducing cell death *ex vivo* of MM cells form 76 patients showed higher sensitivity in samples from patients with t(11:14) than samples from t(11:14)-negative patients (115). MM patients with t(11;14) translocations, who represents 15-20% of MM patients be more sensitive to venetoclax as they have high ratios of BCL-2/MCL-1 in their tumor cells (116, 117). In human myeloma cell lines (HMCLs), a synergism between of compounds individually inhibiting Bcl-2 and Mcl-1 has been confirmed, and the combination of these drugs overcomes Mcl-1 resistance in MM (118, 119). Moreover, mTOR inhibition (everolimus) increases Bcl-2/Mcl-1 ratio in HMCLs by enhancing the binding of transcription factors IKZF3 and Blimp-1 to the BCL2 promoter and therefore enhances venetoclax anti-MM effects (120). Transcriptomic analysis in a panel of 31 myeloma cell lines and 25 patient samples showed an enrichment of specific B-cell genes and specific B cell surface markers, including CD20 and CD79A in venetoclax- sensitive myeloma cells (121). These results could help identifying patients who will respond to treatment with venetoclax. Recently, Todoerti et al (122) showed difference in expression pattern in *BCL2* gene family members between MM cells with t(11:14) compared with MM cells without translocation. There is a need to determine potential predictive biomarkers for venetoclax -sensitive samples. Response to venetoclax correlated with higher *BCL2*:*MCL1* and *BCL2*:*BCL2L1* mRNA expression ratios as confirmed by biomarker analysis (*BCL2* overexpression, *BCL2L1* (coding for Bcl-xL) downregulation); these ratios are essential response predictors for venetoclax and were significantly higher in t(11:14) MM cells compared to MM cells without t(11:14) translocation (116, 123). In addition to Mcl-1, Bcl-xL may be a potential resistance factor to venetoclax (124) as single agent or in combination with bortezomib. The 1q21 gain is also a high-risk biomarker of resistance to venetoclax through the upregulation of MCL-1 (125). Dual targeting of Mcl-1 and Bcl-2 *in vitro* and *ex vivo* increased cell death in resistant cells and showed a synergy between these drugs (126, 127). Functional profiling of BCL2 dependence can also predict clinical response in MM, as an alternative approach to precision medicine that utilizes preclinical BH3 profiling and *ex vivo* testing with venetoclax to determine what level of *ex vivo* drug sensitivity is associated with clinical response (115). Furthermore, disease relapse after treatment resulted in increased NFkB activity and increased *BCL2A1* (coding for BFL-1) as well as Bcl-2 and Bcl-xL (128).

A single-center retrospective study assessing the OS from time of venetoclax refractoriness in MM with t(11;14) or high BCL2 profile showed that the median PFS from the initiation of venetoclax was significantly longer when venetoclax was used as earlier line of treatment (fewer than three prior lines) at 23.2 months vs. 10.4 months in those with three or more prior lines, supporting the use of venetoclax as earlier lines of therapy in t(11:14) MM patients (129). Another study reported that venetoclax sensitivity is correlated to low Electron Transport Chain (ETC) complex I and II activities which propose the use of succinate ubiquinone reductase (SQR) activity as functional biomarker in MM patients for the prediction of venetoclax (130). SQR could be a metabolic target to sensitize resistant MM cells to venetoclax. Venetoclax in patients with t(11;14)(q13;q32) is considered the first example of personalized/precision therapy in the field of MM; suggesting that with more clinical studies this agent will have its place in MM patients management.

Venetoclax has found more success in combination with dexamethasone and bortezomib in comparison to its use as single agent in MM patients (131, 132). Bortezomib inhibits Mcl-1 by stabilizing the Mcl-1-neutralizing protein NOXA (133), dexamethasone up-regulates BCL2 in addition to the proapoptotic Bim, leaving Bcl-2–inhibited cells with an abundance of free Bim (134), therefore the combination of venetoclax, bortezomib, and dexamethasone showed promising effects in R/RMM patients especially those harboring t(11:14) with 78% of partial response (131). In a single-center retrospective study, low dose of venetoclax (≤250mg/day) in combination with DARA, Bortezomib and dexamethasone, 22 RR patients who had received a least 2 lines of prior therapy including at least one PI, showed an overall response rate of 80% in patients harboring t(11:14) vs. 31% in t(11:14)-negative patients, and most importantly, this response was without frequent infection-related serious adverse effects (135). In another phase I multicentric study, the combination of venetoclax, Dara, Dexamethasoe (Ven-Dd in R/RMM t(11:14) patients was compared to the combination of ven, DARA, dexamethasone and bortezomib (Ven-DVd) (cytogenetically unselected patients). The CR was 58% in Ven-Dd group vs. 46% in Ven-DVd group with 33% of patients achieved MRD- vs 21% in Ven-DVd group showing that the addition of DARA with or without bortezomib resulted in deep and durable responses and a higher MRD negativity rate and with no treatment related deaths has been observed in this study (136).

## XPO1 inhibitors in MM

The export of several cargo molecules (proteins, mRNA) is heavily regulated by the protein XPO1, which serves as a mediator of nuclear export by the nuclear pore complex of several potential tumor suppressor proteins and other potential tumor driver proteins such as p53, RB1, and p27 (137). Selinexor is the first XPO1 inhibitor approved by the FDA for used in RRMM in combination with bortezomib after the phase 3 BOSTON trial showed an increased PFS in favor of patients treated with Selinexor bortezomib dexamethasone when compared to bortezomib and dexamethasone (138). Although some preliminary data MM cell lines and patient samples suggest overexpression of E2F1 as a potential mechanism of resistance (139), there is paucity of data on mechanism of resistance in MM to XPO1 inhibitors.

## Conclusions

Our understanding of the genetic landscape of MGUS, SMM, and MM have increased significantly in recent years, resulting in fundamental changes in our model for myeloma development. Furthermore, understanding of the molecular and genetic events associated with a high risk of progression, such as mutations involving the MAPK pathway, *DIS3*, DNA damage repair, and *MYC*, are promising markers that could be incorporated in clinical practice and identify a high-risk subgroup that could benefit from early treatment. Additionally, the presence of biallelic inactivation of *TP53* and 1q amplification identify a subgroup of patients with newly diagnosed MM with poor outcomes, and routine use of this alterations, along with previously defined cytogenetic and clinical models could help refine our stratification models and introduce intense regimens to this patient group. Additionally, the use of CTC’s represents an attractive prognostic biomarker that could be implemented to indicate risk of progression (in the case of MGUS/SMM) and prognosis (for MM), however, global definitions, standardization of the technology used, and cutoffs used are required before this is used routinely in clinical practice.

To this date, targeting t(11;14) or high Bcl2 expression using venetoclax is the most clear example of the target therapy principle in MM, and the worse outcomes in unselected population treated with venetoclax further highlight the selective activity of these targeted agents. As our understanding of the driver events in each subgroup of MM improve and new targeted therapies are developed, the routine identification of these alterations will become more relevant in clinical practice. Similarly, recent molecular studies on the mechanism of resistance to IMiD’s, PI’s, CD38 and BCMA directed therapies have shed light on potential mechanisms of resistance; however, they also show that other mechanisms of resistance are yet to be discovered.

To conclude, the advances in our understanding of the characteristics of MM and precursor conditions have paved the way for the therapeutic and diagnostic advances achieved over the last 20 years. The use of genetic and molecular alterations as prognostic and predictive tools for disease progression and therapeutic response could improve patient care and management. With the increasing affordability of these technologies, we expect they will become an important factor in decision-making in patient care.

## Author contributions

MM, WD, and MB participated in manuscript design and conception. MM and WD elaborated the manuscript and figures. All authors participated in manuscript review and correction.

## Conflict of interest

The authors declare that the research was conducted in the absence of any commercial or financial relationships that could be construed as a potential conflict of interest.

## Publisher’s note

All claims expressed in this article are solely those of the authors and do not necessarily represent those of their affiliated organizations, or those of the publisher, the editors and the reviewers. Any product that may be evaluated in this article, or claim that may be made by its manufacturer, is not guaranteed or endorsed by the publisher.
